# Augmented neutralization of SARS‐CoV‐2 Omicron variant by boost vaccination and monoclonal antibodies

**DOI:** 10.1002/eji.202249841

**Published:** 2022-03-23

**Authors:** Sebastian R. Schulz, Markus Hoffmann, Edith Roth, Katharina Pracht, Deborah L. Burnett, Ohan Mazigi, Wolfgang Schuh, Bernhard Manger, Dirk Mielenz, Christopher C. Goodnow, Daniel Christ, Stefan Pöhlmann, Hans‐Martin Jäck

**Affiliations:** ^1^ Division of Molecular Immunology Department of Internal Medicine 3 Nikolaus‐Fiebiger‐Zentrum Friedrich‐Alexander‐Universität (FAU) Erlangen‐Nürnberg Erlangen Germany; ^2^ Infection Biology Unit German Primate Center‐Leibniz Institute for Primate Research Göttingen Germany; ^3^ Faculty of Biology and Psychology University of Göttingen Göttingen Germany; ^4^ Garvan Institute of Medical Research Sydney New South Wales Australia; ^5^ Faculty of Medicine, UNSW St. Vincent's Clinical School Sydney New South Wales Australia; ^6^ Department of Internal Medicine 3 University Hospital Erlangen Erlangen Bavaria Germany; ^7^ UNSW Cellular Genomics Futures Institute Sydney New South Wales Australia

**Keywords:** boost immunization, coronavirus, COVID‐19, neutralizing antibody, SARS‐CoV‐2, vaccination

## Abstract

Effective vaccines and monoclonal antibodies have been developed against coronavirus disease 2019 (COVID‐19) caused by severe acute respiratory syndrome coronavirus 2 (SARS‐CoV‐2). However, the appearance of virus variants with higher transmissibility and pathogenicity is a major concern because of their potential to escape vaccines and clinically approved SARS‐CoV‐2‐ antibodies. Here, we use flow cytometry‐based binding and pseudotyped SARS‐CoV‐2 neutralization assays to determine the efficacy of boost immunization and therapeutic antibodies to neutralize the dominant Omicron variant. We provide compelling evidence that the third vaccination with BNT162b2 increases the amount of neutralizing serum antibodies against Delta and Omicron variants, albeit to a lower degree when compared to the parental Wuhan strain. Therefore, a third vaccination is warranted to increase titers of protective serum antibodies, especially in the case of the Omicron variant. We also found that most clinically approved and otherwise potent therapeutic antibodies against the Delta variant failed to recognize and neutralize the Omicron variant. In contrast, some antibodies under preclinical development potentially neutralized the Omicron variant. Our studies also support using a flow cytometry‐based antibody binding assay to rapidly monitor therapeutic candidates and serum titers against emerging SARS‐CoV‐2 variants.

## Graphical abstract

Using pseudotype neutralization and flow cytometry‐based spike protein binding assays, Schulz and colleagues show that a boost with BNT162b2 increased neutralizing antibody responses against Omicron, albeit less than against the Wuhan strain and the Delta variant. In contrast, most clinically approved monoclonal antibodies failed to recognize the Omicron variant.

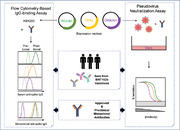

## Introduction

Coronavirus disease 2019 (COVID‐19) caused by the severe acute respiratory syndrome coronavirus 2 (SARS‐CoV‐2) is an infectious disease with variable outcomes. SARS‐CoV‐2 infection, as well as mRNA‐, DNA vector‐, and protein‐based vaccinations, induce neutralizing antibodies and the formation of antigen‐specific T cells for durable cellular and humoral immune memory [1, 2].

Effective vaccines and several potent virus‐neutralizing antibodies have been a game changer in the fight against this devastating COVID‐19 pandemic [3, 4]. The RNA‐based vaccine from BioNTech (BNT162b2) and the two‐antibody cocktail Ronapreve (consisting of *Casirivimab/Imdevimab)* from Regeneron were the first that obtained authorized usage in the European Union (EU) from the European Medicines Agency (EMA) in 2020 for protection against and treatment of early COVID‐19 symptoms, respectively [5]. Furthermore, EMA just authorized the use of a fifth vaccine, the first protein‐based vaccine Nuvaxovid [6], and the anti‐CoV‐2 antibodies Xevudy (Sotrovimab from VirTechnology/GSK) and Regkirona (Regdanvimab from Celltrion) for COVID‐19 treatment [7]. In addition, the two‐antibody cocktail Evusheld (Tixagevimab and Cilgavimab from AstraZeneca) is currently under rolling review [7].

Virus variants of concern have acquired mutations that change the virus’ infectivity and pathogenicity and enable the virus variant to escape at least in part from vaccine‐induced antibody responses and currently approved therapeutic antibodies. For example, sera from double‐vaccinated individuals and therapeutic candidate monoclonal antibodies showed a reduced neutralizing activity against the Delta variant [8, 9]. Here, we use a flow cytometry‐based antibody binding assay with SARS‐CoV‐2 spike‐transfected HEK293 cells and a vesicular stomatitis virus (VSV)‐based pseudotype neutralization assay to analyze whether a third immunization, that is, the second boost vaccination, can increase the serum activity of neutralizing antibodies against the recently emerged and rapidly spreading SARS‐CoV‐2 Omicron variant [10].

## Results and discussion

### Enhanced neutralization of SARS‐CoV‐2 Omicron variant by boosting double‐vaccinated individuals

**Figure 1:**
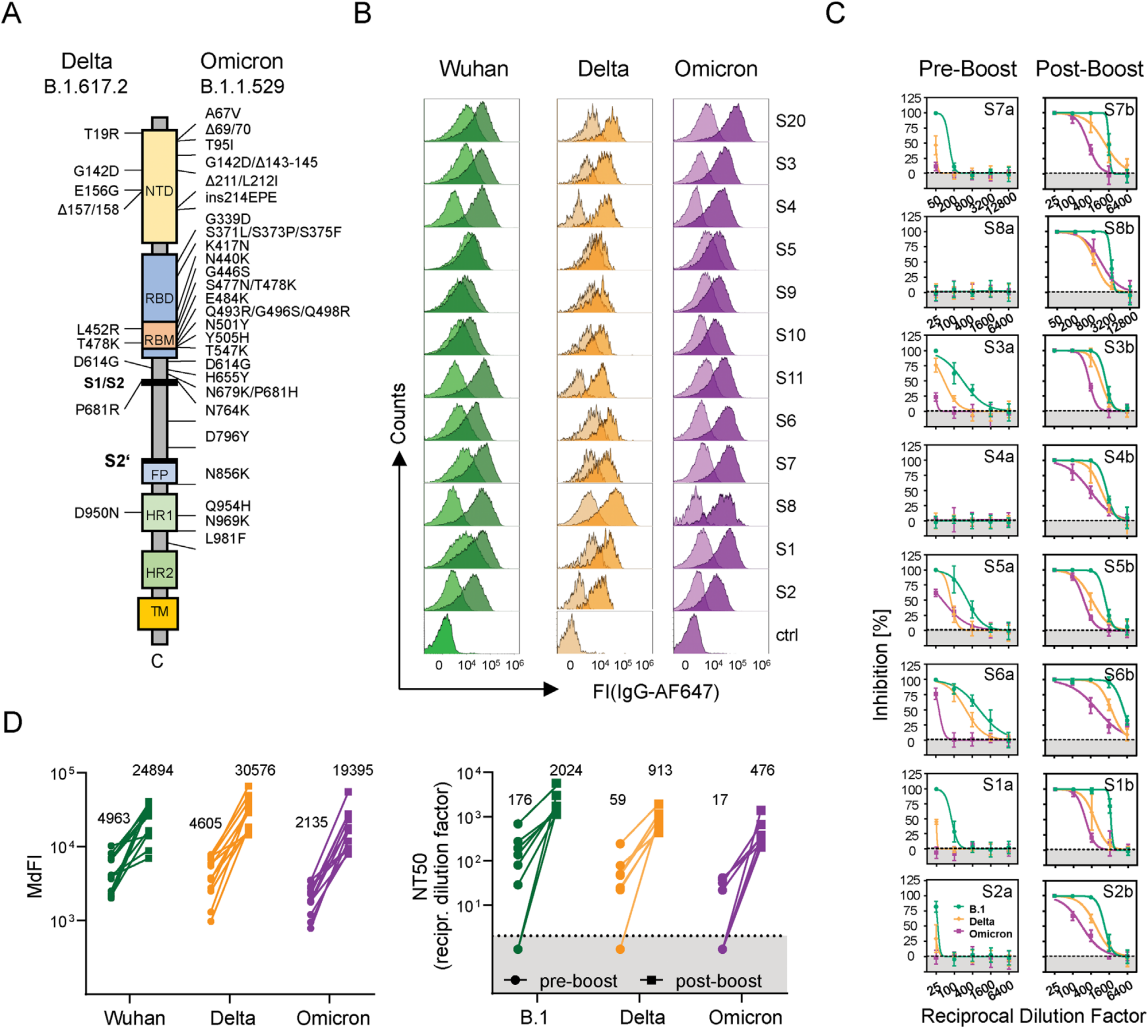
Booster immunization of double‐vaccinated individuals increases Omicron spike protein‐binding and ‐neutralizing antibodies. (A) Schematic overview of the SARS‐CoV‐2 spike proteins. The amino acid changes in the spike proteins of variants of concern (VoC) compared to the reference genome of the Wuhan‐Hu‐1 strain (GenBank accession no. MN908947) are shown for the Delta and Omicron variants. The S1/S2 and S2’ cleavage sites are indicated by black bars. NTD, *N*‐terminal domain; RBD, receptor‐binding domain; RBM, receptor‐binding motif; FP, fusion peptide; HR, heptad repeat, TM, transmembrane domain. (B) Flow cytometry‐based assay to determine antibody binding to SARS‐CoV‐2 spike proteins. HEK293 cells were transiently cotransfected with a SARS‐CoV‐2 spike‐encoding and a GFP‐reporter plasmid. Transfected cells were stained with serum samples from vaccinated individuals (1:100 dilutions) followed by staining with AF647‐conjugated goat antibodies against human IgG. Cells were gated, as depicted in Supporting information Figure S2. Assays were performed independently two to three times for each serum. Serum from a nonvaccinated/noninfected donor served as negative control (ctrl). AF647 fluorescence intensity (FI) determined in the GFP‐gated cell population (see Supporting information Figure S2) was plotted. Lighter and darker shaded histograms correspond to preboost and postboost sera, respectively. Note: S8 and S10 were donors who received AZD1222 as a first vaccine, mRNA‐1273 and BNT162b2 as a second vaccine, respectively, and then with BNT162b2 as a boost. All other volunteers received only the BNT162b2 vaccine shot. Assays were performed independently two to three times for each serum. (C) VSV‐based SARS‐CoV‐2‐pseudotyped neutralization assay. SARS‐CoV‐2 spike protein‐bearing VSV particles were preincubated with serially diluted immune serum before inoculating Vero cells. Spike protein‐driven cell entry was analyzed by measuring the activity of virus‐encoded firefly luciferase in cell lysates. Presented is the average (mean) % inhibition (from four technical replicates per serum) in spike protein‐driven cell entry, which was normalized against a sample without serum (set to 0% Inhibition). Error bars indicate the standard deviation. The SARS‐CoV‐2 B.1 variant differs from the Wuhan‐Hu‐1 stain by the absence of the D614G mutation. (D) Binding intensity correlates with neutralization activity. The median fluorescence intensities (MdFI) of (B) and the neutralization titer 50 (NT_50_, calculated as the reciprocal of the dilution resulting in 50% neutralization) were plotted before (preboost) and after (postboost) the third immunization with BNT162b2. The numbers above the data points indicate the mean of the MdFI and NT50 values. Data points below the dashed line indicate samples for which no neutralizing activity was observed.

**Figure 2:**
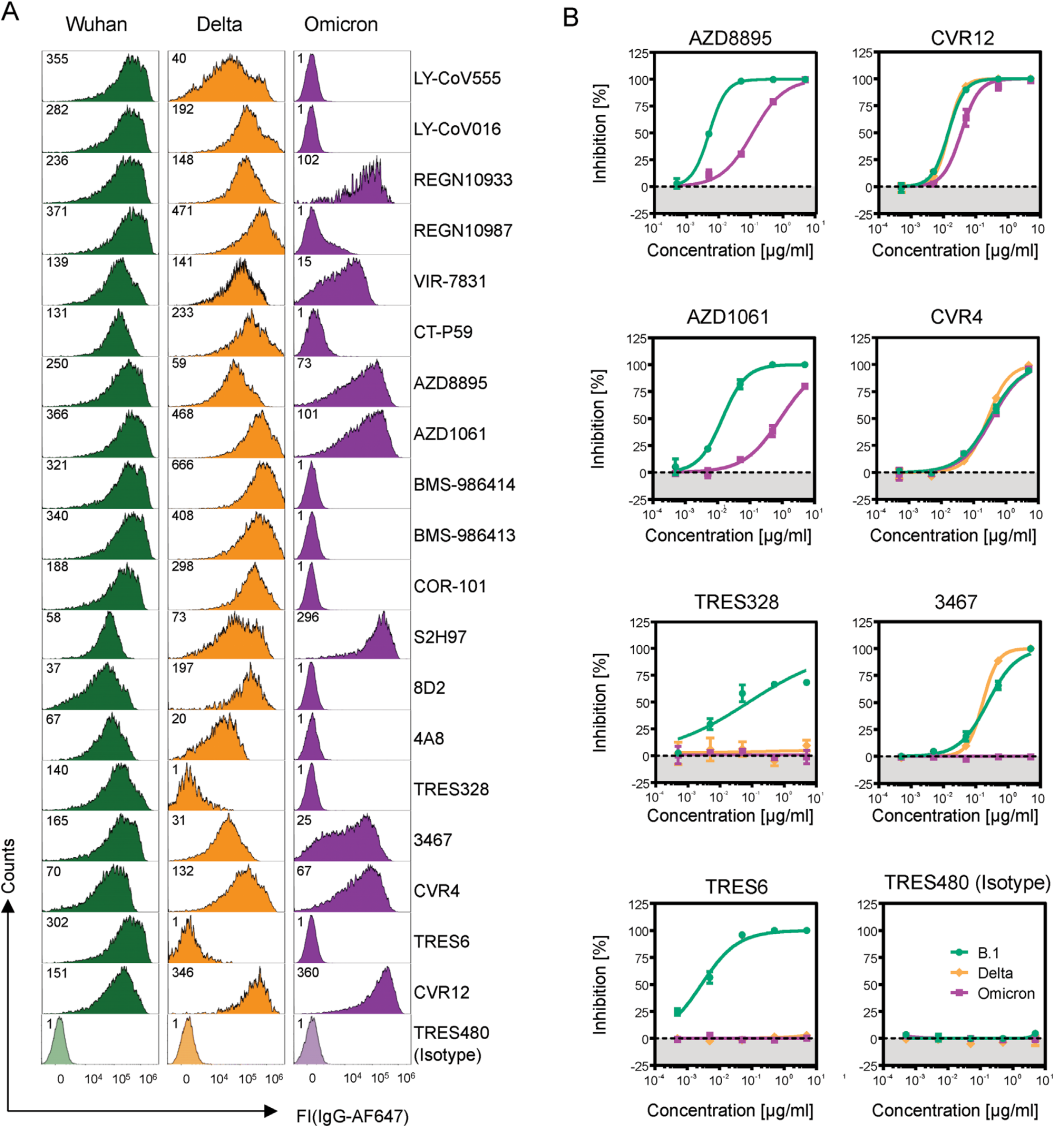
SARS‐CoV‐2 Omicron variant escapes most clinically approved antibodies but can be neutralized by monoclonal antibodies under preclinical development. (A) Flow cytometry‐based assay to determine the binding potential of monoclonal antibodies to SARS‐CoV‐2 spike proteins. The binding of monoclonal antibodies to SARS‐CoV‐2 spike‐transfected HEK293 was determined in a flow cytometer as described in Fig. 1B. Recombinant isotype‐matched TRES480 antibodies served as a negative control. Numbers indicate median fluorescence intensities (MdFI). The assay was performed independently two to three times for each antibody. (B) VSV‐based SARS‐CoV‐2‐pseudotyped neutralization assay. SARS‐CoV‐2 spike protein‐bearing VSV particles were preincubated with serially diluted monoclonal antibodies, and spike protein‐driven cell entry in Vero cells was analyzed as described in Fig. 1C. Presented is the average (mean) % inhibition (from four technical replicates per antibody) in spike protein‐driven cell entry, which was normalized against a sample without antibody (set to 0% Inhibition). Error bars indicate the SD. IC50 values were calculated as the concentration resulting in 50% neutralization and are presented in Table 1.

To test our hypothesis that the third dose of BNT162b2 augments the Omicron spike‐binding and ‐neutralizing antibody response, we recruited individuals who have received a boost vaccination between 4 to 9 months after their second vaccination and collected sera samples before and 10 to 78 days after their third dose (Supporting information Table S1). To quickly determine whether the antibody binding capacity to the Omicron spike protein increases after a boost, we used a flow cytometry‐based IgG‐binding assay with HEK293 cells that were transiently transfected with the spike membrane protein of the Wuhan‐Hu‐1 strain and the SARS‐CoV‐2 Delta and Omicron variants (Fig. 1A and B). The surface abundance of the spike proteins was comparable among the three variants, as demonstrated by a similar binding of the CVR12 antibody that recognizes a shared conserved epitope (Fig. 2A). The slightly lower surface abundance observed for the Wuhan spike protein could be explained by the absence of the D614G mutation reported to increase the membrane spike protein stability [11].

As expected from previous studies, most individuals showed low to very low spike‐specific IgG 4 to 10 months after their second vaccination, indicating a gradual loss of short‐lived vaccine‐induced antibody‐secreting plasma cells. The decrease in antibody binding capacities correlated with lower neutralization activities for viral particles pseudotyped with the spike proteins of the Wuhan, Delta and Omicron stains (Fig. 1C and D). However, after the third vaccine dose (BNT162b2), all analyzed sera samples exhibited increased binding (Fig. 1B) and neutralization activities (Fig. 1C) against all three variants. Again, compared to the Wuhan‐Hu‐1 strain, the neutralizing antibody titers were reduced to about twofold for Delta and fourfold for the Omicron variant. The reduced potential of a boost vaccination to neutralize pseudotyped virus particles bearing the Omicron spike protein could be explained by a significant fraction of reactivated memory B cells recognizing immunodominant epitopes that are altered in the Omicron spike protein. The receptor‐binding domain (RBD) of the Omicron spike protein contains numerous mutations, several of which have been described to mediate antibody evasion [12]. In summary, the observed correlation of SARS‐CoV‐2 pseudovirus neutralization and the IgG binding to the spike protein highlights the practicality of flow cytometry‐based antibody binding assays to quickly assess future SARS‐CoV‐2 variants and monitor the success of vaccination.

### The SARS‐CoV‐2 Omicron variant escapes most clinically approved antibodies

To determine whether the Omicron variant can escape the neutralization by therapeutic antibodies, we screened a collection of clinically approved and preclinically tested candidate antibodies with our flow cytometry‐based antibody‐binding assay (Fig. 2A). Strikingly, most tested antibodies failed to bind to Omicron spike protein, among them clinically approved antibodies from Eli Lilly (Ly‐CoV555 alias Bamlanivimab and Ly‐CoV016 alias Etesevimab), Regeneron (REGN10987 alias Imdevimab), and Celltrion (CT‐P59 alias Regdanvimab), as well as candidate antibodies were currently tested in clinical trials (e.g., STE90‐C11 or COR‐101 from Corat and the two antibodies BMS986414 and 986414 from Bristol Myers Squibb) (Fig. 2A). Of note, VIR‐7831 (alias Sotrovimab) is the only currently approved therapeutic antibody with discernible binding to the Omicron spike (Fig. 2A) and efficient neutralization of Omicron‐pseudotyped particles ([1, 14, 15, 16, 17] and Table 1). However, compared to the B.1 variant (Wuhan‐Hu‐1 spike protein sequence bearing a D614G mutation), Sotrovimab has a two‐ to ‐threefold reduction in its neutralization activity against the Omicron variant, that is, the IC50 value drops from 90 to 260 ng/mL (see Table 1). Therefore, the in vivo efficacy could be reduced, and further studies are required to critically evaluate the full retention of the clinical benefit of Sotrovimab at current dosing.

**Table 1:** Binding and neutralizing activities of clinically and preclinically developed monoclonal antibodies against spike proteins from SARS‐CoV‐2 variants^§^

		Wuhan‐Hu‐1	B.1	DeltaB.1.617.2		OmicronB.1.1.529	
	Antibody(Alias)	MdFI x 10^3^	IC50 ng/mL	MdFI x 10^3^	IC50 ng/mL	MdFI x 10^3^	IC50 ng/mL
**Approved**	**LY‐CoV555** (Bamlanivimab)	355	21 [13] 3 [14]	40	3517 [14]	1	>10 000 [13] >10 000 [15]
	**LY‐CoV016** (Etesevimab)	282	59 [13] 4 [14]	192	4 [14]	1	>10 000 [13] >10 000 [15]
	**REGN10933** (Casirivimab)	236	9 [13] 1 [14]	148	2 [14]	103	>10 000 [13] 1078 [15]
	**REGN10987** (Imdevimab)	371	25 [14] 1 [15]	471	1 [14]	1	>10 000 [13] >10 000 [15]
	**VIR‐7831** (Sotrovimab)	139	91 [13] 8 [14]	141	14 [14]	15	260 [13] 165 [15]
	**CT‐P59** (Regdanivimab)	131	4 [13]	233	ND	1	>10 000 [13]
**Trials**	**AZD8895** (Tixagevimab)	250	5 4 [13]	59	ND	73	115 >10 000 [13]
	**AZD1061** (Cilgavimab)	366	14 8 [13]	468	ND	101	882 2772 [13]
	**BMS‐986414** (C135)	321	ND	666	ND	1	ND
	**BMS‐986413** (C144)	340	ND	408	ND	1	ND
	**STE90‐C11** (COR‐101)	188	ND	298	ND	1	ND
**Preclinical**	**S2H97**	58	280 [13]	74	ND	296	1368 [13]
	**8D2**	37	ND	197	ND	1	ND
	**4A8**	67	ND	20	ND	1	ND
	**TRES328 **	140	79	1	>10 000	1	>10 000
	**3467 **	165	236	31	165	25	>10 000
	**CVR4 **	70	315	132	269	67	368
	**TRES6 **	302	3	1	>10 000	1	>10 000
	**CVR12**	151	14	346	15	360	36

IC50, half maximal inhibitory concentration; MdFI, Median fluorescence intensity.

Values rounded to full numbers.

^§^ More information on antibodies in Supporting information Table S2 and S3.

We also found that neutralizing antibodies against the *N*‐terminal domain (NTD) of the spike protein (e.g., 8D2, 4A8 [16], TRES328, Table 1 and Supporting information Tables S2 and S3) have lost the binding against Delta and Omicron spike protein. As expected, the lack of binding of the anti‐NTD TRES328 antibody correlated with loss of neutralization (Fig. 2B), which is consistent with the accumulation of mutations in the NTD supersite in the variants of concern [12].

Based on the excellent correlation between an antibody's loss to bind and its failure to neutralize SARS‐CoV‐2 variants, we conclude that the flow cytometry‐based antibody binding assay is a valuable tool to quickly prescreen SARS‐CoV‐2 spike variants for antibody evasion, that is, antibodies that have lost their binding potential to the spike protein of a SARS‐CoV‐2 variant will also fail to neutralize the respective virus.

In contrast to published results [17], the antibodies AZD8895 and AZD1061 (aliases Tixagevimab and Cilgavimab) from AstraZeneca and REGN10933 (alias Casirivimab) from Regeneron still bound to the Omicron spike protein in our flow cytometry‐based binding assay. This was surprising because both AZD antibodies still neutralized pseudotyped Omicron particles, albeit with reduced activities (Fig. 2B, Table 1 and [13]), and REGN10933 has completely lost its neutralization activity (Table 1 and [13, 15]). The structural and functional basis for the observed discrepancies between binding and neutralization capabilities of some monoclonal antibodies remains to be solved.

### Identification of SARS‐CoV‐2 Omicron‐neutralizing monoclonal antibodies

We have isolated several SARS‐CoV‐2‐neutralizing antibodies that recognized the spike protein of the Wuhan‐Hu‐1 strain from the human antibody TRIANNI mouse [18, 19], as well as from reconvalescent COVID‐19 patients and human antibody phage display libraries (under review). However, several of the antibodies lost their neutralization potential against the Omicron variant, including the TRIANNI mouse‐derived antibody 3467, that binds to conserved RBD residues away from the binding site of the human angiotensin‐converting enzyme 2, or hACE2 [19]. This is likely mediated by Omicron mutations at positions 371, 373, and 375 in previously highly conserved residues [19]. In contrast, two other antibodies under preclinical development, CVR4 and CVR12, bound Omicron spike protein in the flow cytometry‐based assay (Fig. 2A) and efficiently neutralized pseudotyped Omicron virus particles (Fig. 2B). CVR12 binds to the receptor‐binding motif and blocks the binding of hACE2; CVR4 cobinds with CVR12 and interacts with a region on RBD distant to the ACE2 binding site (under review). Hence, these antibodies represent promising preclinical candidates to treat Omicron‐infected patients either as a single antibody or as a cocktail. This is an important finding, considering that only one approved monoclonal antibody (i.e., Sotrovimab) is currently available for clinical use, and several promising candidates in phase II and III trials (Table 1, and Supporting information Tables S2 and S3) show a negligible or significantly diminished therapeutic potential against the Omicron variant.

## Materials and methods

### Flow cytometry‐based binding assay for SARS‐CoV‐2 spike‐specific antibodies

The flow cytometry‐based binding assay was performed as described by Schuh et al. [20], following the guidelines by Cossarizza et al. [21]. Briefly, HEK293 cells were transiently cotransfected with the SARS‐CoV‐2‐spike‐encoding plasmids (see Supporting information Materials and Methods) combined with a GFP‐encoding plasmid using the PEI method.

Two days after transfection, cells were stained for 10 min on ice with either serum samples from vaccinated individuals in a 1:100 dilution in FACS buffer (PBS supplemented with 0.1% sodium azide and 2% FCS) or monoclonal human IgG1 antibodies (1 μg/mL in FACS buffer). Sera and recombinant antibodies were titrated to maximize the staining index at one fixed antibody concentration (Supporting information Figure S1).

Serum from a nonvaccinated/non‐SARS‐CoV‐2‐infected donor and the human IgG1/κ TRES480 antibody served as negative controls. In addition, bound antibodies were detected with AF647‐conjugated polyclonal goat anti‐human IgG antibodies (Southern Biotech, Birmingham, AL). Cells were gated as depicted in Supporting information Figure S2 and analyzed using a CytoFLEX S flow cytometer (Beckman Coulter).

### Serum samples

Sera from individuals who never tested positive for SARS‐CoV‐2 infection and were double vaccinated with BNT162b2/BNT162b2 (BNT/BNT) or AZD1222/mRNA‐1273 (AZ/MOD) were collected 1 day before or on the day of the third vaccination with BNT162b2 (boost) and 13 to 78 days after the boost. Details on the serum samples are summarized in Supporting information Table S1. The ethical approval (No. 157_20 B) for conducting this analysis was granted by the institutional review board of the University Clinic of Erlangen as the responsible ethics committee for all participating institutions. All human serum samples were obtained with written informed consent from the participants.

### Antibodies

Human recombinant antibodies were Protein G‐purified from supernatants of HEK293 or CHO cells that were transfected with expression vectors encoding the constant domains of the heavy (H) and light (L) chains of human IgG1 (BioIntron, Shanghai, China). The variable sequences of the antibodies were retrieved from appropriate sequence databanks and the literature (Supporting information Table S3). All antibodies had a purity of >95% and contained >98% monomeric IgG as assessed by SDS PAGE and size exclusion chromatography, respectively (not shown).

## Concluding remarks

The encouraging results of this study are that all double‐vaccinated individuals who received a third vaccination (boost vaccination) with BNT162b2 showed an increase in the capacity of serum antibodies to bind to spike protein and neutralize all tested SARS‐CoV‐2 variants, albeit to a lesser degree for the Omicron variant than for the Delta or Wuhan‐Hu‐1 variants. We conclude that the booster vaccination with a SARS‐CoV‐2 strain lacking the mutation of the Omicron variant still increases the amount of Omicron‐neutralizing serum antibodies. Therefore, a booster vaccination is warranted to reduce the risk of severe COVID‐19 symptoms after an Omicron infection as the waning immunity after two doses hardly offers protection against the new variant. This is supported by recent reports from the UK Health Security Agency, the Kaiser Southern California and the Center for Disease Control, USA [22, 23, 24]. All three studies support the conclusion that a third dose reduces COVID‐19‐associated hospitalization by the Omicron variant by 90%.

An intriguing finding of our study is that most of the therapeutic COVID‐19 antibodies with approval from EMA and the FDA are not effective against the spike protein of the Omicron variant (Fig. 2A, Table 1). However, the EMA‐ and FDA‐approved antibody, Sotrovimab, as well as two of the preclinical antibodies developed in our laboratories (CVR4 and CVR12), retain their capacity to bind to the mutated spike protein and neutralize the Omicron variant in pseudotyped assays (Fig. 2A and B, Table 1), warranting further clinical development.

Our findings confirm recently published studies from other groups using sera from vaccinated individuals [25, 26, 27] and monoclonal antibodies [13, 15, 16, 17, 28, 29, 30]. However, few other studies correlated flow cytometry‐based binding and virus neutralization activity of vaccinated individuals (Fig. 1D) or clinically approved antibodies against the Omicron variant [17]. Therefore, the straightforward flow cytometry‐based assay outlined here can be used (1) to rapidly monitor the kinetics of titers of serum antibodies against a presently dominating or an emerging SARS‐CoV‐2 variant of concern (Fig. 1), (2) prescreen clinically approved monoclonal antibodies for treating COVID‐19 infected patients; and (3) evaluate the potential of clinical trials with antibody candidates (Fig. 2 and Table 1).

## Conflict of interest

HMJ is CEO of CoVER Antibodies GmbH and has filed a patent application for the TRES antibodies. The rest of the authors declare no commercial and financial conflict of interest.

## Author contributions

SRS and HMJ designed the study, analyzed, and interpreted the data, and wrote the manuscript. MH and SP designed the study and performed the VSV pseudotype neutralization assays; ER acquired and analyzed data. DB, OM, CCG, and DC provided antibodies 3467, CVR12, and CVR4; WS, KP, and DM contributed to interpreting the results, discussion, and manuscript writing. BM provided human serum samples.

### Peer review

The peer review history for this article is available at https://publons.com/publon/10.1002/eji.202249841.

**Abbreviations**
COVID‐19: Coronavirus disease 2019
EMA: European Medicines Agency
hACE2: human angiotensin‐converting enzyme 2
NTD: *N*‐terminal domain
RBD: receptor‐binding domain
SARS‐CoV‐2: severe acute respiratory syndrome coronavirus
VSV: vesicular stomatitis virus

## Supporting information

Supporting information

## Data Availability

All data required to interpret the data are provided in the main document or the supporting data section.
