# Supplementary material for: Augmented neutralization of SARS‐CoV‐2 Omicron variant by boost vaccination and monoclonal antibodies
Source: Eur J Immunol. 2022 Mar 23;52(6):970–7. doi: 10.1002/eji.202249841 (PMC9087419; doi:10.1002/eji.202249841)
Supplement: Supplementary file 1 — Supporting information [file EJI-52-970-s001.pdf]

SUPPORTING INFORMATION

EJI Schulz et al. 2022

Augmented Neutralization of SARS-CoV-2 Omicron by Boost Vaccination and Monoclonal Antibodies

SUPPORTING METHODS

Plasmids

Pseudotyping

Virus neutralization assay

SUPPORTING FIGURES

**Supporting Figure S1**	Flow cytometry-based titration of SARS-CoV-2 spike protein-binding serum IgG and monoclonal antibodies
**Supporting Figure S2**	Gating strategy for flow cytometry assays.
**Supporting Figure S3.**	Comparison of infectivity by pseudoviruses harboring the spike protein variants.

SUPPORTING TABLES

**Supporting Table S1**	Overview of serum donors.
**Supporting Table S2**	Recombinant human antibodies used in this study.
**Supporting Table S3**	Amino acid sequences of monoclonal antibodies used in this study

SUPPORTING MATERIALS & METHODS

Plasmids

Codon-optimized pCG1-based plasmids encoding the spike proteins of SARS-CoV-2 (Wuhan-Hu-1 Spike protein, GenBank QHD43416.1, AA1-1273), SARS-CoV-2 B.1 (differs from Wuhan-Hu-1 by a D614G mutation), and SARS-CoV-2 B.1.617.2 (Delta) have previously been described [1, 2]. The sequence of the spike protein in the expression vector for B.1.1.529 (Omicron) is based on the isolate hCoV19/Botswana/R40B58_BHP_3321001245/2021 (GISAID Accession ID: EPI_ISL_6640919) and was generated as described. All spikes contain a C-terminal truncation of the last 18 amino acids.

Pseudotyping

Rhabdoviral pseudotypes harboring SARS-CoV-2 S protein were produced as described [3]. Briefly, HEK293T cells were transfected with expression plasmids encoding SARS-CoV-2 spike protein. 24 hours later, cells were infected with a replication-deficient reporter VSV (VSV ∗ ΔG-FLuc) for 1 hour. Residual input VSV was neutralized with a monoclonal antibody against VSV-G, and clarified supernatants were aliquoted and stored at - 80 °C. Pseudoviruses were pre-screened for comparable infectivity (and adjustment of infectivity by dilution, if required) before neutralization experiments were performed to avoid that grossly different infectivity between pseudoviruses may affect data interpretation (Supporting Figure S3).

Virus neutralization assay

Neutralization experiments were performed as previously described [1]. Briefly, SARS-CoV-2 spike protein pseudotyped VSV particles were pre-incubated for 30 min at 37°C with the respective monoclonal antibody or unrelated control human IgG (5, 0.5, 0.05, 0.005, 0.0005 µg/ml) or different dilutions of sera from vaccinated individuals (dilution range: 1:25 to 1:12,800) and added to Vero cells (African green monkey kidney, female, kidney; CRL-1586, ATCC; RRID: CVCL_0574; Further, we thank Andrea Maisner and Gert Zimmer for providing reagents). Particles exposed to medium without antibodies served as control. Transduction efficiency was determined at 16-18 hours post-inoculation by determining luciferase activities in cell lysates, as described [1]. IC50 values were calculated by plotting the neutralization activity in percent against the antibody concentrations and using the normalized response vs. inhibitor equation (variable slope) of GraphPad Prism 7.02.

References for Method Section

1 Hoffmann, M., Krüger, N., Schulz, S., Cossmann, A., Rocha, C., Kempf, A. and Nehlmeier, I. et al., The Omicron variant is highly resistant against antibody-mediated neutralization: Implications for control of the COVID-19 pandemic. Cell. 2021.
2 Arora, P., Sidarovich, A., Krüger, N., Kempf, A., Nehlmeier, I., Graichen, L. and Moldenhauer, A.-S. et al., B.1.617.2 enters and fuses lung cells with increased efficiency and evades antibodies induced by infection and vaccination. Cell reports. 2021. 37: 109825.
3 Kleine-Weber, H., Elzayat, M. T., Wang, L., Graham, B. S., Müller, M. A., Drosten, C., Pöhlmann, S. and Hoffmann, M., Mutations in the Spike Protein of Middle East Respiratory Syndrome Coronavirus Transmitted in Korea Increase Resistance to Antibody-Mediated Neutralization. Journal of virology. 2019. 93.

SUPPORTING FIGURES

A

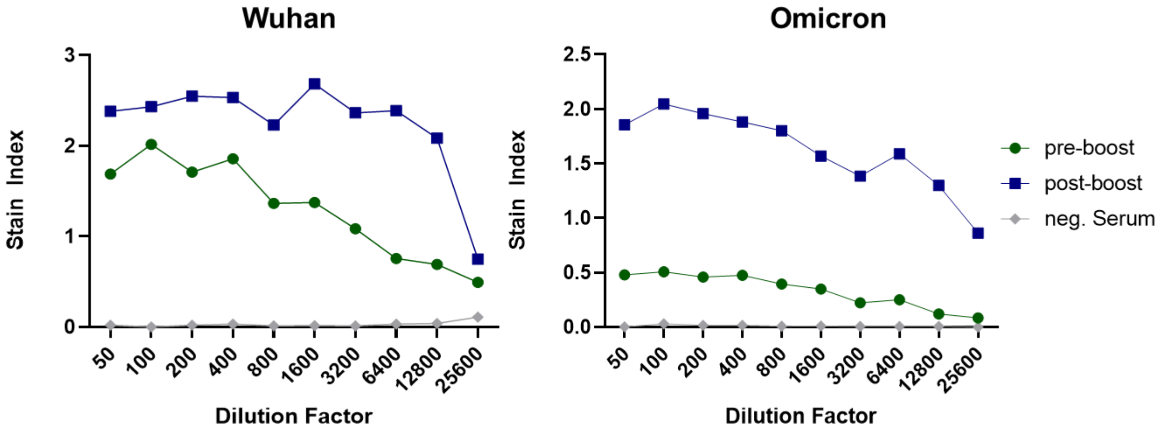

B

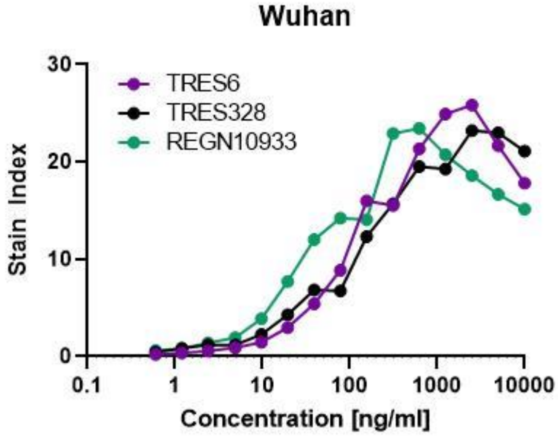

Supporting Figure S1. Flow cytometry-based titration of SARS-CoV-2 spike protein-binding serum IgG and monoclonal antibodies

HEK293 cells co-transfected with spike protein variants (Wuhan/Omicron) and a GFP-encoding reporter plasmid were incubated with **(A)** serial dilutions of a negative control serum (grey) and a pre- (green) and post-boost (blue) serum from the same donor and **(B)** monoclonal human IgG1 antibodies. Antibody binding was detected after secondary staining with anti-human IgG-AF647. The stain index in (A) was calculated for each serum dilution on GFP-positive and –negative cells as described by Bigos (Bigos, M. Separation Index: An Easy-to-Use Metric for Evaluation of Different Configurations on the Same Flow Cytometer. Current Protocols in Cytometry. 2007; 40:1.21.1-1.21.6. DOI: 10.1002/0471142956.cy0121s40)

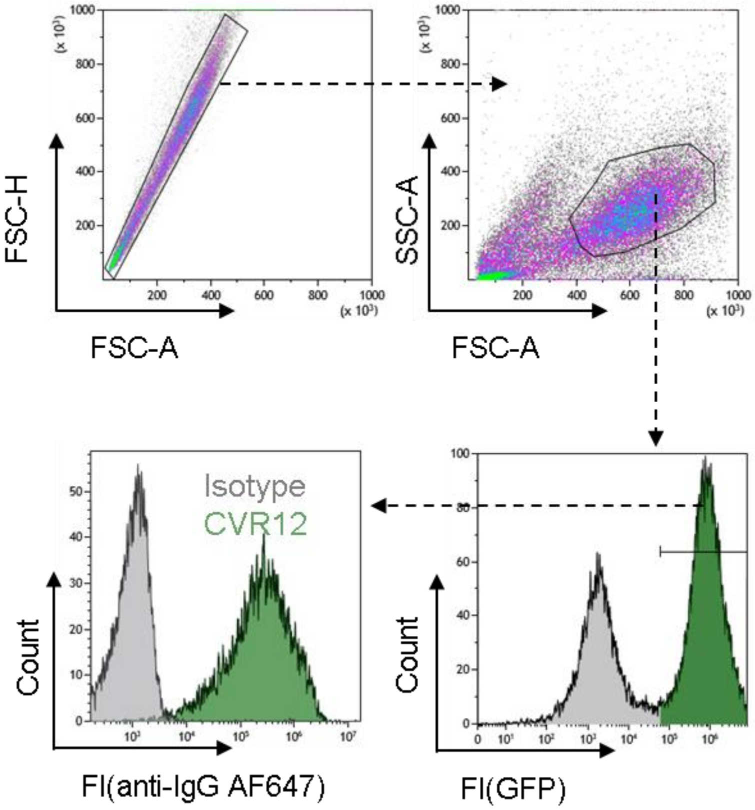

Supporting Figure S2. Exemplary gating strategy for flow cytometry assays

HEK293 cells were transiently co-transfected with a plasmid encoding SARS-CoV-2 variant spike proteins and a GFP expression plasmid. Spike-binding monoclonal antibodies or serum antibodies on co-transfected GFP-positive cells were detected by secondary staining with a goat anti-human IgG-AF647 antibody.

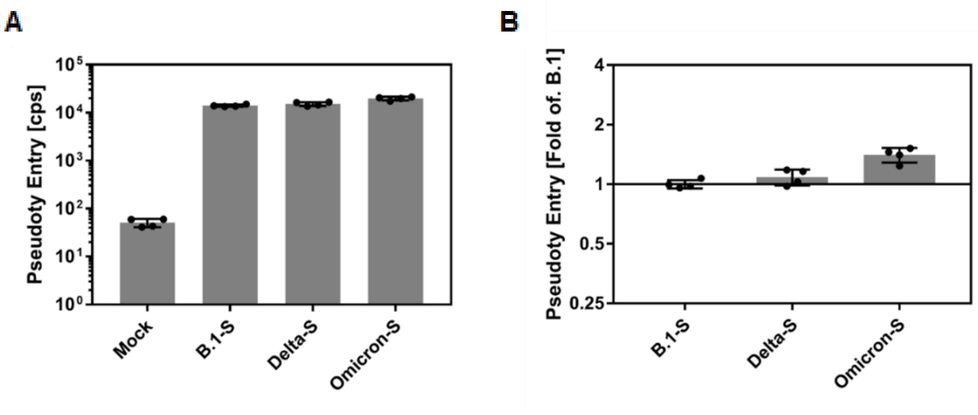

Supporting Figure S3. Comparison of infectivity by pseudoviruses harboring the spike proteins under study.

(A) Infectivity of pseudoviruses used for neutralization experiments. Infectivity was assessed by measuring luciferase activity in Vero cells inoculated with identical volumes of pseudoviruses. Mock-treated (uninfected) cells served as control. Presented are the raw luciferase counts (given as counts per second) from four technical replicates, and error bars indicate the standard deviation. **(B)** Normalized infectivity of pseudoviruses used for neutralization experiments. The normalized luciferase counts are presented in panel A with pseudoviruses bearing B.1-S serving as reference (set as 1). Error bars indicate the standard deviation. S, spike protein.

SUPPORTING TABLES

Supporting Table S1. Overview of Serum Donors.

Serum Donor			Vaccination 1		Vaccination 2		Vaccination 3			Serum Collection		
Code	Sex	Age	Vaccine [1]	Date	Vaccine	Date	Vaccine	Date	mths [2] after 2. vaccination	1	2	days
1	m	66	BNT162b2	22.03.21	BNT162b2	13.04.21	BNT162b2	25.10.21	6,5	19.10.21	25.11.21	37
2	f	65	BNT162b2	28.01.21	BNT162b2	18.02.21	BNT162b2	13.09.21	6,9	13.09.21	06.10.21	23
3	f	36	BNT162b2	28.01.21	BNT162b2	18.02.21	BNT162b2	13.09.21	6,9	13.09.21	06.10.21	23
4	f	38	BNT162b2	26.01.21	BNT162b2	16.02.21	BNT162b2	13.09.21	7,0	13.09.21	06.10.21	23
5	f	25	BNT162b2	26.01.21	BNT162b2	16.02.21	BNT162b2	13.09.21	7,0	14.09.21	06.10.21	22
6	f	33	BNT162b2	28.01.21	BNT162b2	18.02.21	BNT162b2	13.09.21	6,9	13.09.21	06.10.21	23
7	m	26	BNT162b2	03.01.21	BNT162b2	24.01.21	BNT162b2	29.10.21	9,3	29.10.21	25.11.21	27
8	m	27	AZD1222	28.02.21	mRNA-1273	09.05.21	BNT162b2	12.11.21	6,2	12.05.21	28.11.21	200
9	m	29	BNT162b2	25.04.21	BNT162b2	05.06.21	BNT162b2	05.10.21	4,1	05.10.21	22.12.21	78
10	f	27	AZD1222	25.02.21	BNT162b2	01.05.21	BNT162b2	02.11.21	6,2	02.11.21	23.12.21	51
11	f	63	BNT162b2	06.04.21	BNT162b2	18.05.21	BNT162b2	30.11.21	6,5	04.12.21	22.12.21	18
20	m	22	BNT162b2	25.05.21	BNT162b2	06.07.21	BNT162b2	07.12.21	5,1	09.12.21	22.12.21	13

[1] Code names were used throughout the publication; [2] months

Supporting Table S2. Recombinant human antibodies used in this study.

Antibody				Reactivity	Class	Status	Developer
Name	Generic name	Brand name	Other name				
**LY-CoV555**	Bamlanivimab			RBD	II [1, 2]	EUA	Eli Lilly
**LY-CoV016**	Etesevimab		Shi-CB6	RBD	I [1]	EUA	Eli Lilly
**REGN10933**	Casirivimab	Ronapreve (REGN2)		RBD	I [1]	EUA	Regeneron
**REGN10987**	Imdevimab			RBD	III [2, 3]	EUA	Regeneron
**VIR-7831**	Sotrovimab	Xevudy	S309	RBD	III [2, 3]	EUA	Vir Biotechnology / GlaxoSmithKline
**CT-P59**	Regdanvimab	Regkirona		RBD	I [1]	EUA	Celltrion
**AZD8895**	Tixagevimab	Evusheld		RBD	I	Phase III	Astra Zeneca
**AZD1061**	Cilgavimab			RBD	II	Phase III	Astra Zeneca
**BMS-986414**			C135	RBD	III [2, 3]	Phase II	Bristol Myers Squibb
**BMS-986413**			C144	RBD	II [2, 3]	Phase II	Bristol Myers Squibb
**STE90-C11**			COR-101	RBD		Phase I/II	CORAT
**S2H97**				RBD	V [4]	Pre-clinical	
**8D2**				NTD	NTD	Pre-clinical	
**4A8**				NTD	NTD	Pre-clinical	
**TRES328**				NTD	NTD	Pre-clinical	
**3467**				RBD	IV	Pre-clinical	
**CVR4**				RBD	-	Pre-clinical	Under review
**TRES6**				RBD	I	Pre-clinical	
**CVR12**				RBD	-	Pre-clinical	Under review

1 **Kumar et al.** Current status of therapeutic monoclonal antibodies against SARS-CoV-2. *PLoS pathogens*. 2021. **17**: e1009885.

2 **Shrestha et al.**, Broadly-Neutralizing Antibodies Against Emerging SARS-CoV-2 Variants. *Frontiers in immunology*. 2021. **12**: 752003.

3 **Barnes et al.** Structural classification of neutralizing antibodies against the SARS-CoV-2 spike receptor-binding domain suggests vaccine and therapeutic strategies. *bioRxiv the preprint server for biology*. 2020.

4 **Starr et al.** SARS-CoV-2 RBD antibodies that maximize breadth and resistance to escape. *Nature*. 2021. **597**: 97–102.

Supporting Table S3. Amino acid sequences of monoclonal antibodies used in this study

Antibody	Mature Ig amino acid sequence		Reference	
	VH	VL	Publication	VH/VL Sequence Accession
**LY-CoV555**	QVQLVQSGAEVKKPGSSVKVSCKASGGTFSNYAISWVRQAPGQGLEWMGRIIPILGIANYAQKFQGRVTITADKSTSTAYMELSSLRSEDTAVYYCARGYYEARHYYYYYAMDVWGQGTAVTVSS	DIQMTQSPSSLSASVGDRVTITCRASQSISSYLSWYQQKPGKAPKLLIYAASSLQSGVPSRFSGSGSGTDFTLTITSLQPEDFATYYCQQSYSTPRTFGQGTKVEIK	[1]	PDB: 7L3N_D/7L3N_E
**LY-CoV016**	EVQLVESGGGLVQPGGSLRLSCAASGFTVSSNYMSWVRQAPGKGLEWVSVIYSGGSTFYADSVKGRFTISRDNSMNTLFLQMNSLRAEDTAVYYCARVLPMYGDYLDYWGQGTLVTVSS	DIVMTQSPSSLSASVGDRVTITCRASQSISRYLNWYQQKPGKAPKLLIYAASSLQSGVPSRFSGSGSGTDFTLTISSLQPEDFATYYCQQSYSTPPEYTFGQGTKLEIK	[2]	PDB: 7C01_C/7C01_D
**REGN10933**	QVQLVESGGGLVKPGGSLRLSCAASGFTFSDYYMSWIRQAPGKGLEWVSYITYSGSTIYYADSVKGRFTISRDNAKSSLYLQMNSLRAEDTAVYYCARDRGTTMVPFDYWGQGTLVTVSS	DIQMTQSPSSLSASVGDRVTITCQASQDITNYLNWYQQKPGKAPKLLIYAASNLETGVPSRFSGSGSGTDFTFTISGLQPEDIATYYCQQYDNLPLTFGGGTKVEIK	[3]	PDB: 6XDG_B/6XDG_D
**REGN10987**	QVQLVESGGGVVQPGRSLRLSCAASGFTFSNYAMYWVRQAPGKGLEWVAVISYDGSNKYYADSVKGRFTISRDNSKNTLYLQMNSLRTEDTAVYYCASGSDYGDYLLVYWGQGTLVTVSS	QSALTQPASVSGSPGQSITISCTGTSSDVGGYNYVSWYQQHPGKAPKLMIYDVSKRPSGVSNRFSGSKSGNTASLTISGLQSEDEADYYCNSLTSISTWVFGGGTKLTVL	[3]	PDB: 6XDG_C/6XDG_A
**VIR-7831**	QVQLVQSGAEVKKPGASVKVSCKASGYPFTSYGISWVRQAPGQGLEWMGWISTYQGNTNYAQKFQGRVTMTTDTSTTTGYMELRRLRSDDTAVYYCARDYTRGAWFGESLIGGFDNWGQGTLVTVSS	EIVLTQSPGTLSLSPGERATLSCRASQTVSSTSLAWYQQKPGQAPRLLIYGASSRATGIPDRFSGSGSGTDFTLTISRLEPEDFAVYYCQQHDTSLTFGGGTKVEIK	[4]	PDB: 6WPS_F/6WPS_G
**CT-P59**	QITLKESGPTLVKPTQTLTLTCSFSGFSLSTSGVGVGWIRQPPGKALEWLALIDWDDNKYHTTSLKTRLTISKDTSKNQVVLTMTNMDPVDTATYYCARIPGFLRYRNRYYYYGMDVWGQGTTVTVSS	ELVLTQPPSVSAAPGQKVTISCSGSSSNIGNNYVSWYQQLPGTAPKLLIYDNNKRPSGIPDRFSGSKSGTSATLGITGLQTGDEADYYCGTWDSSLSAGVFGGGTELTVL	[5]	PDB: 7CM4_H/7CM4_L
**AZD8895**	QMQLVQSGPEVKKPGTSVKVSCKASGFTFMSSAVQWVRQARGQRLEWIGWIVIGSGNTNYAQKFQERVTITRDMSTSTAYMELSSLRSEDTAVYYCAAPYCSSISCNDGFDIWGQGTMVTVSS	EIVLTQSPGTLSLSPGERATLSCRASQSVSSSYLAWYQQKPGQAPRLLIYGASSRATGIPDRFSGSGSGTDFTLTISRLEPEDFAVYYCQHYGSSRGWTFGQGTKVEIK	[6]	PDB: 7L7D_H/7L7D_L
**AZD1061**	EVQLVESGGGLVKPGGSLRLSCAASGFTFRDVWMSWVRQAPGKGLEWVGRIKSKIDGGTTDYAAPVKGRFTISRDDSKNTLYLQMNSLKTEDTAVYYCTTAGSYYYDTVGPGLPEGKFDYWGQGTLVTVSS	DIVMTQSPDSLAVSLGERATINCKSSQSVLYSSNNKNYLAWYQQKPGQPPKLLMYWASTRESGVPDRFSGSGSGAEFTLTISSLQAEDVAIYYCQQYYSTLTFGGGTKVEIK	[6]	PDB: 7L7E_C/7L7E_D

**BMS-986414**	QVQLVESGGGVVQPGRSLRLSCAASGFTFSSYAMHWVRQAPGKGLEWVAVIPFDGRNKYYADSVTGRFTISRDNSKNTLYLQMNSLRAEDTAVYYCASSSGYLFHSDYWGQGTLVTVSS	DIQMTQSPSTLSASVGDRVTITCRASQSISNWLAWFQQKPGKAPKLLIYEASSLESGVPSRFSGSGSGTEFTLTISSLQPDDFATYYCQQYNSYPWTFGQGTKVEIK	[7]	PDB: 7K8Z_H/7K8Z_L
**BMS-986413**	EVQLVESGGGLIQPGGSLRLSCAASGFTVSNNYMSWVRQAPGKGLEWVSVIYSGGSTYYADSVKGRFTISRDKSKNTLYLQMNRLRAEDTAVYYCAREGEVEGYNDFWSGYSRDRYYFDYWGQGTLVTVSS	QSALTQPASVSGSPGQSITISCTGTSSDVGGYNYVSWYQQHPGKAPKLMIYDVSNRPSGVSNRFSGSKSGNTASLTISGLQAEDEADYYCSSYTSSSTRVFGTGTKVTVL	[7]	PDB: 7K90_H/7K90_L
**STE90-C11**	QVQLVESGGGLVQPGGSLRLSCAASGLTVSSNYMSWVRQAPGKGLEWVSVIYSGGSTYYADSVKGRFTISRDDSKNTLYLQMNSLRAEDTAVYYCARDVADAFDIWGQGTMVTVSS	DIVMTQSPSFLSASVGDRVTITCRASQGISSYLAWYQQKPGKAPKLLIYAASTLQSGVPSRFSGSGSGTEFTLTISSLQPEDFATYYCQQLNSYPPFTFGPGTKVDIK	[8]	PDB: 7B3O_H/7B3O_L
**S2H97**	QVRLVQSGAEVKKSGESLKISCKGSGYSFTSYWIGWVRQMPGKGLEWMGIIYPGDSDTRYSPSFQGQVTISADKSISTVYLQWSSLKASDTAMYYCARQWSHYTYDYYYWGQGTLVTISS	QSVLTQPASVSGSPGQSITISCTGISSDVGGYNSVSWYQQHPGKAPKLMIYDVTNRPSGVSNRFSGSKSGNTASLTISGLQAEDEADYYCSSYTSSSTPPYVFGTGTKVSVL	[9]	PDB: 7M7W_C/7M7W_D
**8D2**	EVQLVESGGGLVQPGGSLRLSCAASGFTFSSYWMSWVRQAPGKGLEWVANINQDGSEKYYVDSVKGRFTISRDNAKNSLYLQVNSLRAEDTAVYYCARDWDYDILTGSWFGAFDIWGQGTTVTVSS	DIQMTQSPSSLSASVGDRVTITCRASQGIRNDLGWYQQKPGKAPKRLIYAASSLQSGVPSRFSGSGSGTEFTLTISSLQPEDFATYYCLQHNSYPLTFGGGTKVEIK	[10]	PDB: 7DZX_H/7DZX_L
**4A8**	EVQLVESGAEVKKPGASVKVSCKVSGYTLTELSMHWVRQAPGKGLEWMGGFDPEDGETMYAQKFQGRVTMTEDTSTDTAYMELSSLRSEDTAVYYCATSTAVAGTPDLFDYYYGMDVWGQGTTVTVSS	EIVMTQSPLSSPVTLGQPASISCRSSQSLVHSDGNTYLSWLQQRPGQPPRLLIYKISNRFSGVPDRFSGSGAGTDFTLKISRVEAEDVGVYYCTQATQFPYTFGQGTKVDIK	[11]	PDB: 7C2L_H/7C2L_L
**TRES328**	QVHLVQSGAEVKKPGASVKVSCKVSGYTLVEVSVHWVRQAPGKGFEWMGGFDPENAATIYAQKFQGRVTMTEDTSTDTAYMELSSLRYEDTAVYFCATAPAVAGPLYYYYYGMDVWGQGTTVTVSS	DIVMTQTPLSSPVTLGQPASISCRSSQSLVHSDGNTYLSWLQQRPGQPPRLLIYKVSNRFSGVPDRFSGSGAGTEFTLKISRVEAEDVGVYYCTQATQFPTFGQGTKLEIK	[12]	GenBank: QXI90082.1/QXI90083.1
**3467**	QVQLQESGPGLVKPSETLSLTCTVSGGSISSYHWNWIRQPPGKGLEWIGYIYYSGNTNYNPSLKSRVSISTDTSKNQFSLKLSSVTAADTAVYYCVREMRRGYSGYDYWDLYAFDIWGQGTMVTVSS	DIQLTQSPSFLSASVGDRVTITCRASQGISSYLAWYQQKPGKAPNLLIYAASTLQSGVPSRFSGSGSGTEFTLTISSLQPEDFATYYCQQLNSYPHTFGQGTKLEIK	[13]	PDB: 7MSQ_H/7MSQ_L
**CVR4**	-	-	Under review	
**TRES6**	QVQVVESGGGVVQPGRSLRLSCAASGFTFSSYGMHWVRQAPGKGLEWVAVIWYDGSNKYYADSVKGRFTISRDNSKNTLYLQMNSLRVEDTAVYYCVRETVDGMDVWGQGTTVTVSS	NIQMTQSPSAMSASVGDSVTITCRARQDINNYLAWFQQKPGKVPKHLIYAASSLLSGVPSRFSGSGSGTEFTLTISSLQPEDFATYYCLQHNSYPYTFGQGTKLEIK	[12]	GenBank: QXI90072.1/QXI90073.1
**CVR12**	-	-	Under review	

Supporting Table S3 (continuation). Amino acid sequences of monoclonal antibodies used in this study

Antibody Sequence References

1. Jones BE, Brown-Augsburger PL, Corbett KS, Westendorf K, Davies J, Cujec TP, Wiethoff CM, et al. The neutralizing antibody, LY-CoV555, protects against SARS-CoV-2 infection in nonhuman primates. Science Translational Medicine. 2021.

2. Shi R, Shan C, Duan X, Chen Z, Liu P, Song J, Song T, et al. A human neutralizing antibody targets the receptor-binding site of SARS-CoV-2. Nature. 2020; 584:120–124

3. Hansen J, Baum A, Pascal KE, Russo V, Giordano S, Wloga E, Fulton BO, et al. Studies in humanized mice and convalescent humans yield a SARS-CoV-2 antibody cocktail. Science. 2020.

4. Pinto D, Park Y-J, Beltramello M, Walls AC, Tortorici MA, Bianchi S, Jaconi S, et al. Cross-neutralization of SARS-CoV-2 by a human monoclonal SARS-CoV antibody. Nature. 2020; 583:290–295

5. Kim C, Ryu D-K, Lee J, Kim Y-I, Seo J-M, Kim Y-G, Jeong J-H, et al. A therapeutic neutralizing antibody targeting receptor binding domain of SARS-CoV-2 spike protein. Nat Commun. 2021; 12:288

6. Zost SJ, Gilchuk P, Case JB, Binshtein E, Chen RE, Nkolola JP, Schäfer A, et al. Potently neutralizing and protective human antibodies against SARS-CoV-2. Nature. 2020; 584:443–449

7. Robbiani DF, Gaebler C, Muecksch F, Lorenzi JCC, Wang Z, Cho A, Agudelo M, et al. Convergent antibody responses to SARS-CoV-2 in convalescent individuals. Nature. 2020; 584:437–442

8. Bertoglio F, Führer V, Ruschig M, Heine PA, Abassi L, Klünemann T, Rand U, et al. A SARS-CoV-2 neutralizing antibody selected from COVID-19 patients binds to the ACE2-RBD interface and is tolerant to most known RBD mutations. Cell Reports. 2021; 36:109433

9. Starr TN, Czudnochowski N, Liu Z, Zatta F, Park Y-J, Addetia A, Pinto D, et al. SARS-CoV-2 RBD antibodies that maximize breadth and resistance to escape. Nature. 2021; 597:97–102

10. Liu Y, Soh WT, Kishikawa J, Hirose M, Nakayama EE, Li S, Sasai M, et al. An infectivity-enhancing site on the SARS-CoV-2 spike protein targeted by antibodies. Cell. 2021; 184:3452-3466

11. Chi X, Yan R, Zhang J, Zhang G, Zhang Y, Hao M, Zhang Z, et al. A neutralizing human antibody binds to the N-terminal domain of the Spike protein of SARS-CoV-2. Science. 2020

12. Peter AS, Roth E, Schulz SR, Fraedrich K, Steinmetz T, Damm D, Hauke M, et al. A pair of noncompeting neutralizing human monoclonal antibodies protecting from disease in a SARS-CoV-2 infection model. European Journal of Immunology

13. Burnett DL, Jackson KJL, Langley DB, Aggrawal A, Stella AO, Johansen MD, Balachandran H, et al. Immunizations with diverse sarbecovirus receptor-binding domains elicit SARS-CoV-2 neutralizing antibodies against a conserved site of vulnerability. Immunity. 2021; 54:2908-2921
